# Magnesium Toxicity in an Obstetric Patient Due to Preeclampsia-Related Renal Dysfunction Despite Administration of a Standard Pritchard Regimen: A Case Report

**DOI:** 10.7759/cureus.101584

**Published:** 2026-01-15

**Authors:** Ndapewoshali L Pinehas, Ambrose Rukewe, Onochie U Nweze

**Affiliations:** 1 Department of Anaesthesia, Windhoek Central Hospital, Windhoek, NAM; 2 Department of Anaesthesia, Katutura State Hospital, Windhoek, NAM; 3 Division of Anaesthesiology, University of Namibia, Windhoek, NAM

**Keywords:** acute kidney injury, emergency cesarean section, intensive care management, magnesium sulfate toxicity, pre-eclampsia

## Abstract

Magnesium toxicity could occur in the treatment of preeclampsia/eclampsia, especially when the patient has coexisting renal impairment. If undetected and promptly treated, hypermagnesemia can cause severe fetomaternal complications and death.

We present the case of a 22-year-old pregnant woman at 32 weeks of gestation, who was referred to our hospital with eclampsia, treated with intramuscular magnesium sulfate following a standard Pritchard regimen. An emergency cesarean section was carried out under general anesthesia due to multiorgan dysfunction. Following the cesarean delivery, she was successfully managed with mechanical ventilation and hemodialysis.

## Introduction

Magnesium sulfate is the first-line treatment for seizure prophylaxis in severe preeclampsia and the control of seizures associated with eclampsia. Despite having a low therapeutic index, hypermagnesemia in obstetric patients is rare, but some case reports have described severe complications, including deaths from overdose or under-excretion caused by renal impairment [1-3].

We present this case of magnesium toxicity in an eclamptic patient with renal insufficiency, as a complication treated with one of the recommended magnesium sulfate regimens (Pritchard), to highlight the need for cautious administration and close monitoring in low-resource settings.

## Case presentation

A 22-year-old female patient (gravida 4 and para 3) at 32 weeks of gestation was referred to our hospital with a diagnosis of eclampsia complicated by hemolysis, elevated liver enzymes, and low platelets (HELLP syndrome). She was taken to the local health center after two episodes of seizure at home. Upon arrival at the center, she had another clonic-tonic seizure, and her blood pressure (BP) was 167/106 mmHg, pulse rate 91/min, random blood sugar was 8.8 mmol/L, and urine output via urethral catheter was 500 mL. Magnesium sulfate was administered using the Pritchard regimen (a loading dose of 4 g of magnesium sulfate in 200 mL saline intravenously over 10 minutes, followed by intramuscular injections of 5 g of magnesium sulfate in each buttock, making a total of 14 g). The patient was a known chronic hypertensive who had defaulted on methyldopa for two years. Sublingual nifedipine 10 mg and oral methyldopa 250 mg eight hourly were added. After being stabilized for 2 hours 30 minutes, she was transferred to a nearby district hospital.

Upon arrival at the district hospital, BP was 154/110 mmHg, pulse rate 85, and urine dipstick revealed protein ++. Intramuscular hydralazine 6.25 mg was administered, but the BP remained uncontrollable. Magnesium sulfate was continued at a dose of 5 g intramuscularly four hourly on alternate buttocks; she received four doses totaling 20 g. Altogether, she got 34 g of magnesium sulfate (14 g at the health center and 20 g at the district hospital). She was said to be in the latent phase of labor, with one contraction in 10 minutes lasting 26 seconds; vaginal examination revealed a 1 cm dilated cervix, 50% effaced, station level -1, and intact membranes. The fetal assessment on ultrasound revealed an alive, singleton, intrauterine pregnancy with good cardiac activity, cephalic presentation, estimated fetal weight 1.674 kg, and gestational age 31 weeks and 2 days. The patient was then transferred to the Windhoek Central Hospital.

**Figure 1:**
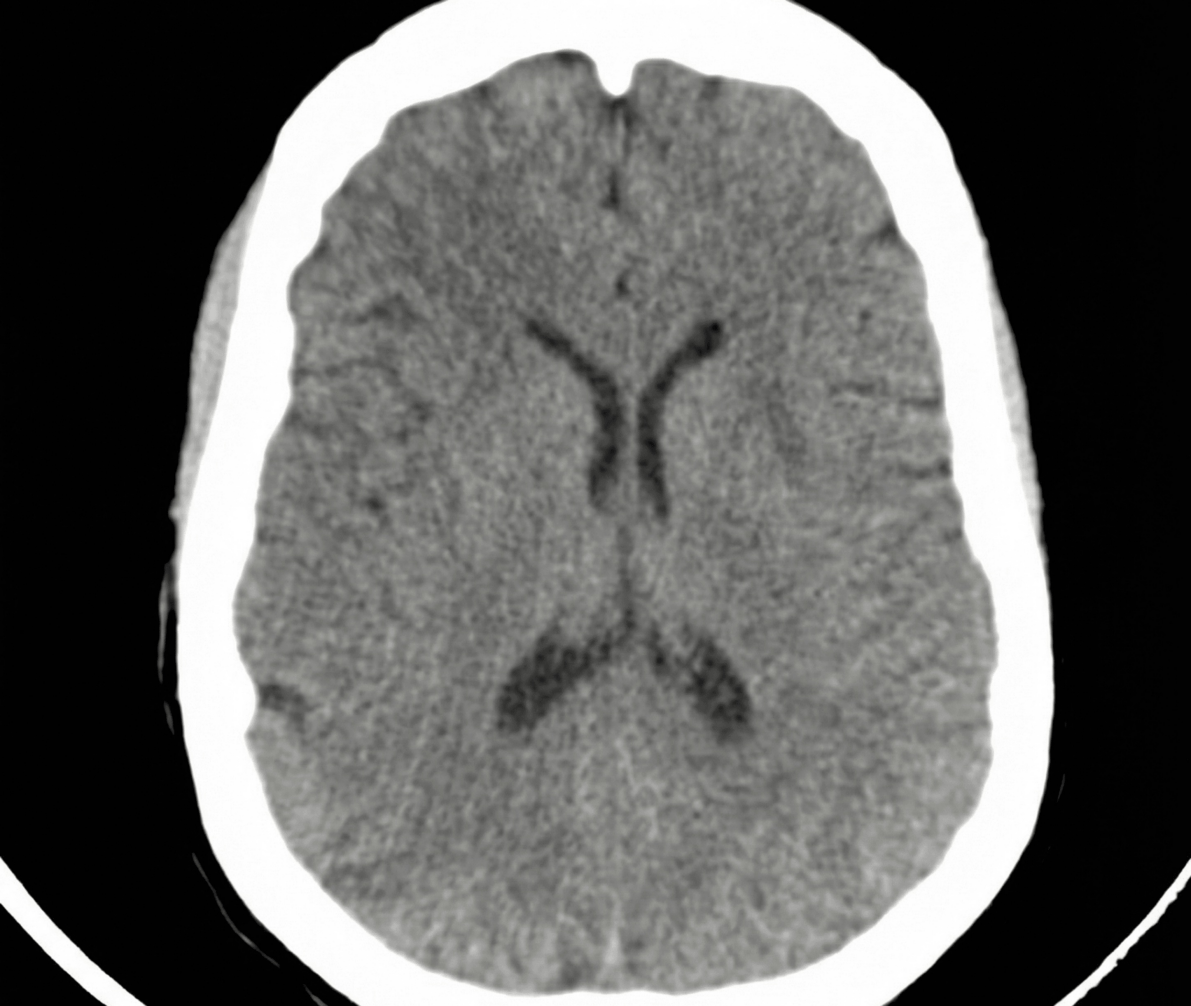
Computed tomography brain indicative of posterior reversible encephalopathy syndrome (PRES)

On examination at our hospital, the patient appeared ill-looking, with altered consciousness (Glasgow Coma Scale (GCS) was 12/15; E = 3, V = 4, and M = 5) and complete loss of patella reflex (which corresponds to zero on a standard deep tendon reflex scale). Her cardiovascular and respiratory system examination was unremarkable (BP was 129/69 mmHg, pulse rate was 97/min, respiratory rate was 20/min, and SaO₂ averaged less than 92% on room air). She was oliguric (700 mL/24 hours), and her urine was dark brown in color. Her laboratory workup results showed elevated levels of urea (14.6 mmol/L), creatinine (311 μmol/L), magnesium (>3.9 mmol/L), alanine transaminase (229 IU/L), aspartate transaminase (717 IU/L), and low platelet count of 37 × 10⁹/L (Table 1). The CT scan of the brain (Figure 1) was suggestive of posterior reversible encephalopathy syndrome (PRES), with no signs of hemorrhage or acute ischemic injury. The diagnosis of eclampsia with HELLP syndrome, acute kidney injury (based on rising serum creatinine levels and significant oliguria (<0.5 mL/kg/hr)), and magnesium toxicity was made. The immediate management involved the administration of 10% intravenous calcium gluconate, 10 mL slowly over 10 minutes. Calcium gluconate was repeated eight hours later. Abdominal ultrasound reported both kidneys as slightly hyperechoic, with reduced corticomedullary differentiation; the right kidney measured 9.7 × 4.0 cm, and the left kidney measured 11.0 × 5.8 cm. No focal lesion, hydronephrosis, or perinephric collections bilaterally; the urinary bladder was empty; uterus normal, no obvious pelvic collections; liver normal, gallbladder with sludge inside; no ductal dilatation or features of cholecystitis; spleen normal; no ascites. No electrocardiography (ECG) was done.

**Table 1:** Laboratory workup results of the patient showing severe multisystemic disorder ALP, Alkaline phosphatase; ALT, Alanine transferase; AST, Aspartate transferase

Variable	On admission	Day 1	Day 2	Day 3	Day 4	Day 5	Day 6	Day 7	Post-discharge	Reference range
Potassium (mmol/L)	3.5	3.3	4.0	4.6	4.3	5.1	5.7	4.9	3.4	3.5-5.1
Sodium (mmol/L)	138	139	139	135	133	134	136	138	142	136-145
Urea (mmol/L)	14.6	15.7	18.7	23.9	15.8	23.2	27.8	25.4	6.0	2.9-8.2
Creatinine (μmol/L)	311	349	457	567.7	453	668	773	649	55.4	64-108
Calcium (mmol/L)	-	2.27	2.06	2.38	2.20	2.34	2.22	2.16	1.76	2.1-2.6
Phosphate (mmol/L)	-	2.68	2.26	2.32	1.40	1.83	1.76	0.85	0.33	0.8-1.5
Magnesium (mmol/L)	-	>3.9	>3.9	>3.9	1.9	1.88	1.82	1.34	1.16	0.7-1.0
Total protein (g/dL)	-	56	44	48	44	49	45	52	61	6.0-8.3
Albumin (g/dL)	-	24	21	20	17	17	15	17	25	3.4-5.5
Bilirubin (mg/dL)	-	99	36	14	10	9	9	8	16	0.1-1.2
ALT (IU/L)	-	194	155	128	91	79	63	57	69	10-40
AST (IU/L)	-	352	193	101	62	66	72	55	79	10-35
ALP (IU/L)	-	153	133	157	165	203	236	288	342	30-130
Hemoglobin (g/dL)	8.8	8.4	7.2	7.3	7.1	6.9	-	6.6	9.4	>11
Platelets (×10⁹/L)	37	68	67	83	100	139	244	382	435	150-400

The decision to deliver the baby operatively was made 16 hours after admission due to multi-organ dysfunction secondary to eclampsia. An emergency cesarean section was performed under standard endotracheal general anesthesia, without muscle relaxant technique, which lasted about 30 minutes. Intraoperatively, the patient was hemodynamically stable, with BPs ranging 108/56-120/64 mmHg, pulse rate 86-92/min, and SaO₂ 96%-99%. The estimated blood loss was 300 mL. A 1.945 kg male baby was delivered, with an Apgar score of 2/10 at 1 minute, 6/10 at 5 minutes, and 8/10 at 10 minutes following resuscitation by the attending neonatologist. The baby was then transferred to the neonatal intensive care unit (NICU) for respiratory support and observation.

The patient remained intubated, admitted to the ICU, and ventilated on pressure-controlled synchronized intermittent mandatory ventilation (PC-SIMV) mode, as the arterial blood gas (ABG) showed metabolic acidosis without respiratory compensation (Table 2). On days 1-3, the laboratory workup (Table 1) revealed worsening oliguria and deteriorating liver and renal functions (rising creatinine levels and liver enzymes), but the serum magnesium was reported to be >3.9 mmol/L (our laboratory did not report the peak magnesium level over the three days). Four more doses of calcium gluconate were administered. She had two sessions of hemodialysis on days 3 and 4, after which her serum magnesium levels reduced to 1.8 mmol/L, and urine output improved.

**Table 2:** Arterial blood gas results of the patient before and after ventilatory support

Variable	Before ventilatory support	After ventilatory support	Reference range
pH	6.8	7.34	7.35-7.45
pCO_2 _(mmHg)	61.7	19.5	35-45
pO_2 _(mmHg)	93	140	75-100
HCO_3 _(mmol/L)	11.3	10.4	22-29
O_2 _saturation (%)	90.4	96.2	95-100
Lactate (mmol/L)	2.49	1.9	0.5-2.2

The patient was successfully weaned off the mechanical ventilator and discharged from the ICU on day 7, in a stable clinical condition, with normal renal function and no neurological deficit.

## Discussion

This case report highlights the importance of careful administration of magnesium sulfate and close monitoring of patients treated for severe preeclampsia with co-existing renal compromise. Hypermagnesemia often results from accidental overdose in the absence of kidney disease, or, as in this index case, from under-excretion due to preeclampsia-related renal dysfunction [1,4-6]. It was significant that this patient had a positive history of chronic hypertension and two years of non-compliance with antihypertensive medication.

Preeclampsia, a pregnancy-associated hypertension and proteinuria, has a 2%-8% global prevalence and is a significant cause of fetomaternal morbimortality, especially in low- and middle-income countries (LMICs). Magnesium sulfate is the drug of choice for prophylaxis of eclampsia in patients with severe preeclampsia and the treatment of eclampsia [7-9]. The patient received 34 g of magnesium sulfate in 24 hours, which was within the recommended daily dose range according to the Pritchard regimen. A healthy kidney would excrete at least 90% of this dose during the first 24 hours; this was not possible in our patient. The Zuspan regimen (4 g intravenous injection over 10-15 minutes, followed by 1 g per hour for 24 hours), with a relatively lower overall dose, would be less hazardous. The total dose in 24 hours in the Dhaka regimen is marginally less by 3 g than Zuspan’s. Furthermore, a study found a 12-hour modification of the Zuspan regimen with about 40% less overall dose, non-inferior to the standard 24-hour treatment [3,10,11]. According to Agarwal et al., low-dose regimens are effective, reduce the risk of magnesium toxicity, and are advisable in low-resource settings where serum magnesium assays are not feasible [12]. However, the ease of intramuscular injections in rural health centers, like the one this patient was taken to after the seizures, makes the Pritchard regimen popular [13].

In our patient, we could not determine the peak serum magnesium level, as the laboratory result on days 1-3 was >3.9 mmol/L despite worsening oliguria, and ABG, which depicted severe metabolic acidosis without respiratory compensation, along with rising serum creatinine concentrations indicating deteriorating renal function. If an ECG evaluation had been done, it would have flagged magnesium toxicity, as a prolonged PR interval and widened QRS are early warning signs. However, her cloudy sensorium, loss of patella reflex, and need for ventilatory support (ostensibly from muscle weakness) were suggestive of moderate to severe magnesemia - an estimate up to or more than 5 mmol/L based on these clinical features, in the absence of actual measurement, since the toxic effects are related to serum magnesium levels. This brings into focus the failure of our laboratory to report the exact serum level. The administration of intravenous calcium gluconate probably reduced the risk of cardiac arrhythmias [5]. The decision for cesarean delivery was beneficial in view of maternal multiorgan dysfunction, but the low Apgar score at birth might be due to elevated ionized magnesium levels from placental transfer, causing hypotonia that necessitated resuscitation and NICU admission [14]. We cannot confirm this in the neonate, since the neonatal magnesium level was not measured. It is noteworthy that the severity of magnesium toxicity in this patient was hampered by obvious laboratory limitations, such as a lack of ECG, no available peak serum magnesium level, and inconclusive ABG results, in keeping with our low-resource practice environment.

Her admission into the ICU for ventilatory support and hemodialysis restored the physiologic magnesium levels and renal function in keeping with the international guideline that hemodialysis treatment is required if kidney function is impaired [15]. Failure to correct the toxic magnesium levels would have worsened the respiratory compromise and led to cardiac arrest, which was avoided in this case.

## Conclusions

In a low-resource setting, consider a lower magnesium sulfate dose that would prevent or manage eclampsia, and prompt hemodialysis should be employed if renal impairment co-exists. Routine clinical and laboratory workups of patients treated with magnesium sulfate should include assessment of patella reflexes, respiratory rate, ECG, blood biochemistry, and ABG.

## References

[1] Kumar K, Al Arebi A, Singh I (2013). Accidental intravenous infusion of a large dose of magnesium sulphate during labor: a case report. J Anaesthesiol Clin Pharmacol.

[2] Richards A, Stather-Dunn L, Moodley J (1985). Cardiopulmonary arrest after the administration of magnesium sulphate. A case report. S Afr Med J.

[3] Lu JF, Nightingale CH (2000). Magnesium sulfate in eclampsia and pre-eclampsia: pharmacokinetic principles. Clin Pharmacokinet.

[4] McDonnell NJ, Muchatuta NA, Paech MJ (2010). Acute magnesium toxicity in an obstetric patient undergoing general anaesthesia for caesarean delivery. Int J Obstet Anesth.

[5] Cavell GF, Bryant C, Jheeta S (2015). Iatrogenic magnesium toxicity following intravenous infusion of magnesium sulfate: risks and strategies for prevention. BMJ Case Rep.

[6] Smith JM, Lowe RF, Fullerton J, Currie SM, Harris L, Felker-Kantor E (2013). An integrative review of the side effects related to the use of magnesium sulfate for pre-eclampsia and eclampsia management. BMC Pregnancy Childbirth.

[7] Bilano VL, Ota E, Ganchimeg T, Mori R, Souza JP (2014). Risk factors of pre-eclampsia/eclampsia and its adverse outcomes in low- and middle-income countries: a WHO secondary analysis. PLoS One.

[8] Osungbade KO, Ige OK (2011). Public health perspectives of preeclampsia in developing countries: implication for health system strengthening. J Pregnancy.

[9] American College of Obstetricians and Gynecologists' Task Force (2013). Hypertension in pregnancy. Obstet Gynecol.

[10] Kattah A (2020). Preeclampsia and kidney disease: deciphering cause and effect. Curr Hypertens Rep.

[11] Grillo EO, Awonuga DO, Dedeke IO (2023). Comparison of Zuspan regimen and its 12-hour modification in women with severe pre-eclampsia and eclampsia in two hospitals in Abeokuta. Pregnancy Hypertens.

[12] Agarwal S, Gupta R, Pandey K (2020). Is low dose magnesium sulfate regimen a better option for treatment of hypertensive disorders of pregnancy: our experience at tertiary care centre. Int J Clin Obstetr Gynaecol.

[13] Akbar MI, Yoseph D, Bachnas MA (2020). Magnesium intoxication in women with preeclampsia with severe features treated with magnesium sulfate. Hypertens Pregnancy.

[14] Abbassi-Ghanavati M, Alexander JM, McIntire DD, Savani RC, Leveno KJ (2012). Neonatal effects of magnesium sulfate given to the mother. Am J Perinatol.

[15] Zipes DP, Camm AJ, Borggrefe M (2006). ACC/AHA/ESC 2006 guidelines for management of patients with ventricular arrhythmias and the prevention of sudden cardiac death: a report of the American College of Cardiology/American Heart Association Task Force and the European Society of Cardiology Committee for practice guidelines (writing committee to develop guidelines for management of patients with ventricular arrhythmias and the prevention of sudden cardiac death): developed in collaboration with the European Heart Rhythm Association and the Heart Rhythm Society. Circulation.

